# Dog-bite injuries in Korea and risk factors for significant dog-bite injuries: A 6-year cross-sectional study

**DOI:** 10.1371/journal.pone.0210541

**Published:** 2019-02-21

**Authors:** Joong Wan Park, Do Kyun Kim, Jae Yun Jung, Se Uk Lee, Ikwan Chang, Young Ho Kwak, Soyun Hwang

**Affiliations:** 1 Department of Emergency Medicine, Seoul National University Hospital, Seoul, Korea; 2 Department of Emergency Medicine, Kangwon National University College of Medicine, Chuncheon, Gangwon-do, Korea; Medical University Graz, AUSTRIA

## Abstract

**Introduction:**

An accurate understanding of the current status of dog-bite injuries in Korea is essential for establishing preventive strategies. There have been no national reports about dog-bite injuries in Korea. This study investigated dog-bite injuries in Korea that were registered in the nationwide injury surveillance database and analysed the risk factors for significant dog-bite injury.

**Methods:**

A multicentre cross-sectional study was conducted using the emergency department (ED)-based Injury In-depth Surveillance (EDIIS) registry in Korea between 2011 and 2016. We defined significant injury as death, admission, surgery, or fracture or amputation. A multivariable logistic regression model was used to obtain the adjusted odds ratios (aORs) for the factors associated with significant dog-bite injuries.

**Results:**

Among 1,537,617 injured patients, 9,966 (6.5 per 1,000 injured patients) presented to the ED for dog-bite injuries (5.6 in 2011 to 7.6 in 2016, *P* for trend < 0.001), and 489 (4.9%) were significant injuries. In the age-specific analysis, there were increasing trends only among teenagers (12−18 years) and adults (> 18 years). Being elderly (≥ 60 years) (aOR: 2.70, 95% CI: 2.15−3.39), having injuries to multiple anatomic sites (aOR: 4.37, 95% CI: 2.96−6.45), being bitten outdoors (aOR: 2.71, 95% CI: 2.20−3.34), and being bitten by a relative’s dog (aOR: 2.37, 95% CI: 1.09−5.17) were strongly associated with significant dog-bite injury.

**Conclusion:**

Dog-bite injuries are increasing in Korea, especially in teenagers and adults. A relative’s or neighbour’s dog may be more dangerous than a stranger’s dog. Preventive strategies are needed to prevent dog-bite injuries in adults and children.

## Introduction

As the proportion of households with companion animals has increased, the number of animal-bite injuries has also increased. In the United States, animal bites account for 1% of all emergency department (ED) visits [1]. Dogs are responsible for the vast majority of animal bites [2]. Dog bites can cause traumatic damage to the tendons and nerves, disability, infections, such as rabies, psychological and emotional trauma, hospitalization and rarely death [3–7].

The proportion of families with companion animals in Korea has increased from 17.4% in 2010 to 21.8% in 2015 according to the Ministry of Agriculture, Food, and Rural Affairs [8]. While several dog-bite events have been reported to the media and there has been growing interest in dog bites in recent years, no nationwide epidemiological report about dog-bite injuries has yet been made in Korea. The lack of systematic data makes it difficult to identify the associated risk factors and to establish effective preventive strategies against dog-bite injury.

In this study, we investigated dog-bite injuries in Korea that were registered in the nationwide injury surveillance database between 2011 and 2016. The present study also analysed the risk factors for significant dog-bite injury, including fracture, amputation or death, and the need for surgery or hospitalization.

## Materials and methods

### Ethics statement

This study was approved by the Institutional Review Board of Seoul National University Hospital (IRB No. 1807-079-958). Informed consent was waived, and patient information was anonymized prior to analysis.

### Study design and patients

This cross-sectional study was conducted using data from the ED-based Injury In-depth Surveillance (EDIIS) registry in Korea. Among all cases registered in the EDIIS registry from January 2011 to December 2016, patients who were bitten by a dog were included in this study as the study population. Dog bite-related injuries were first identified according to the injury mechanism (dog bite), and one researcher then reviewed the injury narratives. After reviewing the injury narratives, if the injuries were caused by bites of animals other than dogs or the injuries were not bites (e.g., dog scratches), they were excluded from the study. If the disposition of emergency care was “transferred out”, the cases were excluded from the study population because of the possibility of duplicate registration.

### National injury registry

The EDIIS has been established by the Korea Centers for Disease Control and Prevention (KCDC) with five hospitals since 2006. The EDIIS was developed to collect and investigate in-depth data on injury mechanisms and causes of injured patients in the ED and to provide information useful for establishing strategies for injury prevention. Since 2010, the number of participating hospitals has increased to 20, and data are currently being collected from patients who have been injured in 23 EDs in Korea. Based on the dataset of the International Classification of External Causes of Injuries (ICECI) by the World Health Organization, the EDIIS database has 246 variables, including patient demographics, injury-related data, prehospital records, clinical findings, diagnosis and medical treatment in the ED, ED disposition, and patient outcomes after admission. Primary surveillance data were collected by physicians from each hospital by checking the box for each variable in the standardized EDIIS registry. Emergency physicians and trained coordinators at each hospital regularly reviewed and revised the recorded information. The completed data were regularly uploaded into a web-based database system of the KCDC by trained coordinators. The quality management committee reviewed the data monthly and provided regular feedback to coordinators for quality improvement [9].

### Variables and measurements

We collected information on patient age and gender, the anatomical site of the injury, wound characteristics, surgery, the place where the accident occurred, reported dog owners, the dates and times of the ED admission and discharge, disposition after emergency care, the dates and times of the hospital admission and discharge, the number of days hospitalized and the outcome of the hospital admission. The patients were divided into the following age groups based on human development and growth to describe the epidemiological characteristics of each age group: infant (< 1 year), toddler (1–3 years), preschooler (4–6 years), school-age (7–12 years), teenager (13–18 years), and adult (> 18 years). The anatomical sites of dog-bite injuries were categorized as head and neck (including the face), torso (including the thorax, abdomen, back, pelvis, and genitals), upper extremity (including the shoulders, upper arms, elbows, forearms, wrists, and hands), lower extremity (hips, thighs, knees, lower legs, ankles and feet) and multiple locations (a combination of one or more of the above anatomic sites) according to the International Classification of Diseases 10^th^ Revision (ICD-10) code and the review of the injury narratives. The wound characteristics were classified as superficial, open, fracture, amputation and other according to the ICD-10 code. The places where the accident occurred were classified as follows: home (including home garden, apartment parking area, and playground at the apartment site), road, commercial facilities, countryside/sea/river, public cultural facilities, and other/unknown. The injury locations were also divided into indoor or outdoor places. The date of ED admission was used to consider monthly variation and 2 photoperiods, namely, long day (April-September) and short day (October-March). Dog owners were classified as follows: household, relative, neighbour/friend, and stranger.

### Main outcomes

The primary outcome of the study was risk factors for significant dog-bite injury. We defined significant injury as patient death, hospitalization, surgery, or diagnosis of fracture or amputation. Mortality was defined as death in the ED or during hospitalization and determined at discharge from the ED or hospital. The secondary outcome of this study was epidemiologic characteristics of dog-bite injury in Korea.

### Statistical analysis

All statistical analyses were performed using STATA version 14.2 (StataCorp LP, College Station, TX, USA). Continuous variables were presented as medians with interquartile ranges, and categorical variables were presented as frequencies with proportions. To assess significant differences between the outcome groups, we used the Wilcoxon rank sum test for continuous variables and the Chi-square test or Fisher’s exact test for categorical variables. The odds ratio (OR) with 95% confidence interval (CI) was calculated by multivariable logistic regression analysis to evaluate the associated factors for significant dog-bite injury. The OR was adjusted for the patient’s age group (0–12 years, 13–18 years, 19–59 years, and ≥ 60 years), anatomical site (head and neck, torso, upper extremity, lower extremity, multiple, and unknown), injury location (indoor, outdoor, and unknown), and dog owner (household, relative, neighbour/friend, stranger, and unknown). The level of statistical significance was defined as a *P* value less than 0.05.

## Results

### Characteristics of dog-bite injury in Korea

**Fig 1:**
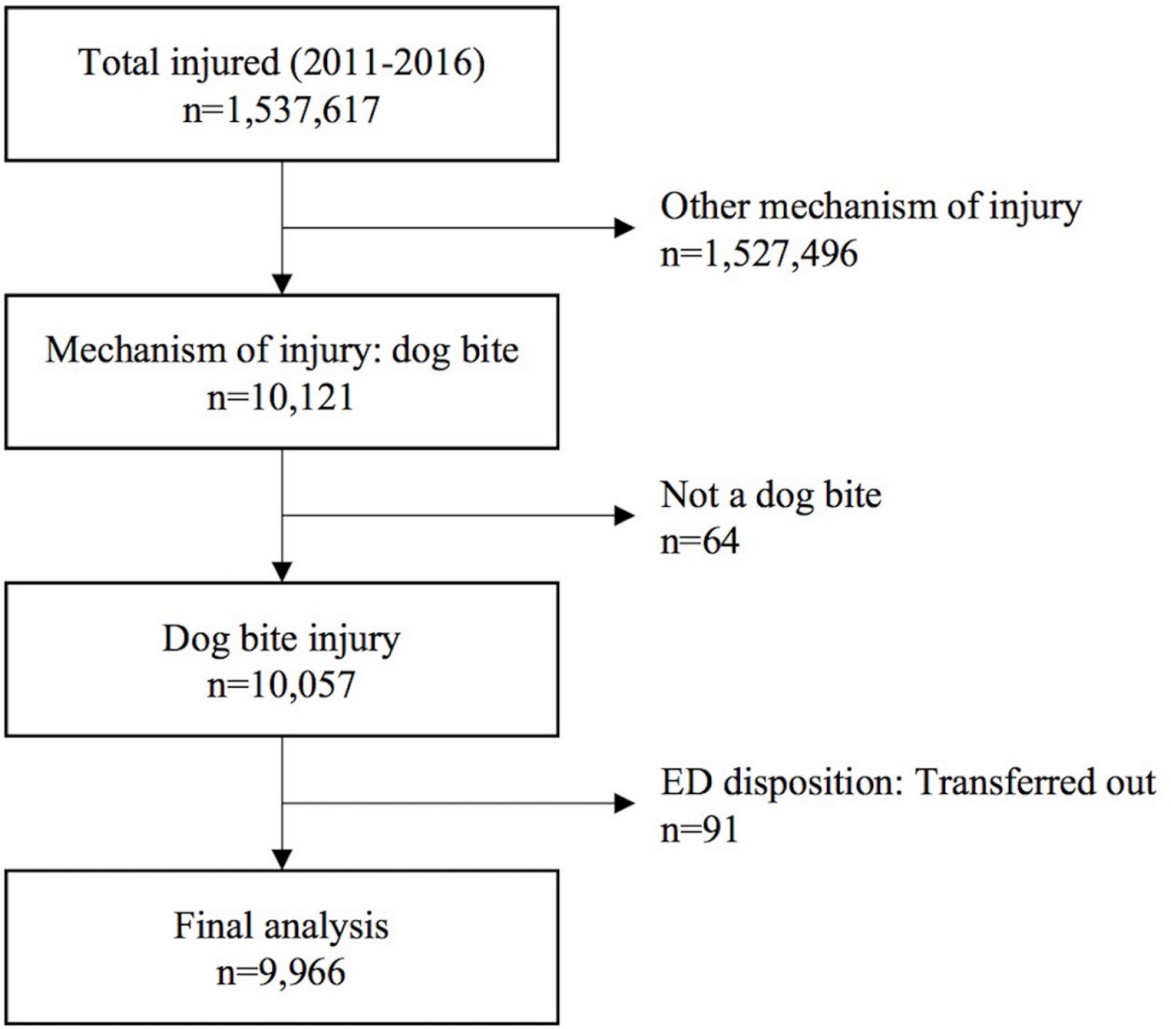
Flow diagram of the study population.

A total of 1,537,617 injured patients were registered in the EDIIS registry from 2011 to 2016. Among them, 10,121 patients were categorized as dog-bite injuries according to the injury mechanism. After a review of the injury narratives, 64 patients were excluded because their injury mechanisms were incorrectly coded. A total of 9,966 (0.6%) patients were included in the final analysis, except for 91 patients who were transferred to other hospitals after emergency care (Fig 1).

**Fig 2:**
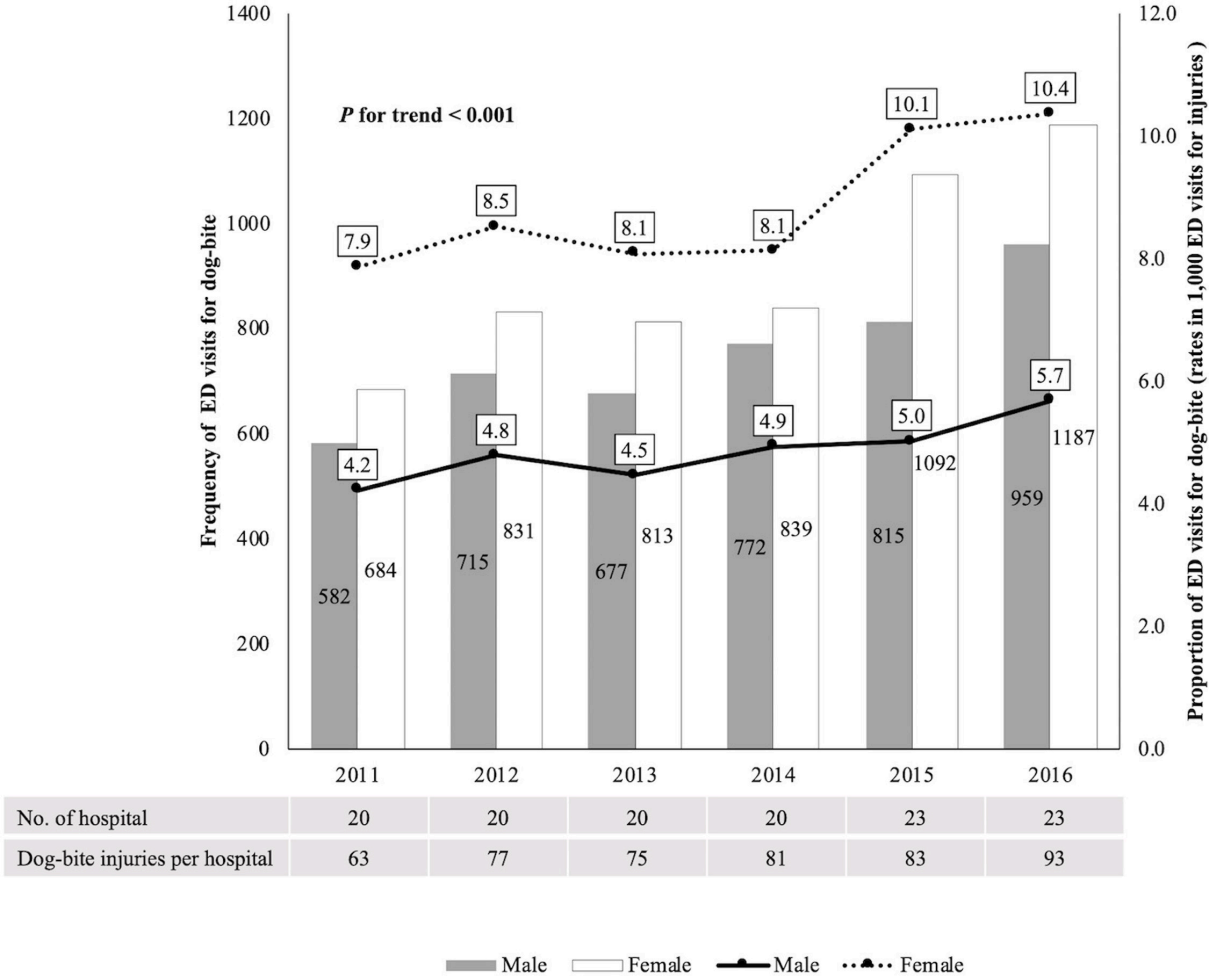
The trends of dog-bite injuries from 2011 to 2016. Both males and females showed increases in the rate of dog-bite injury from 2011 to 2016.

**Fig 3:**
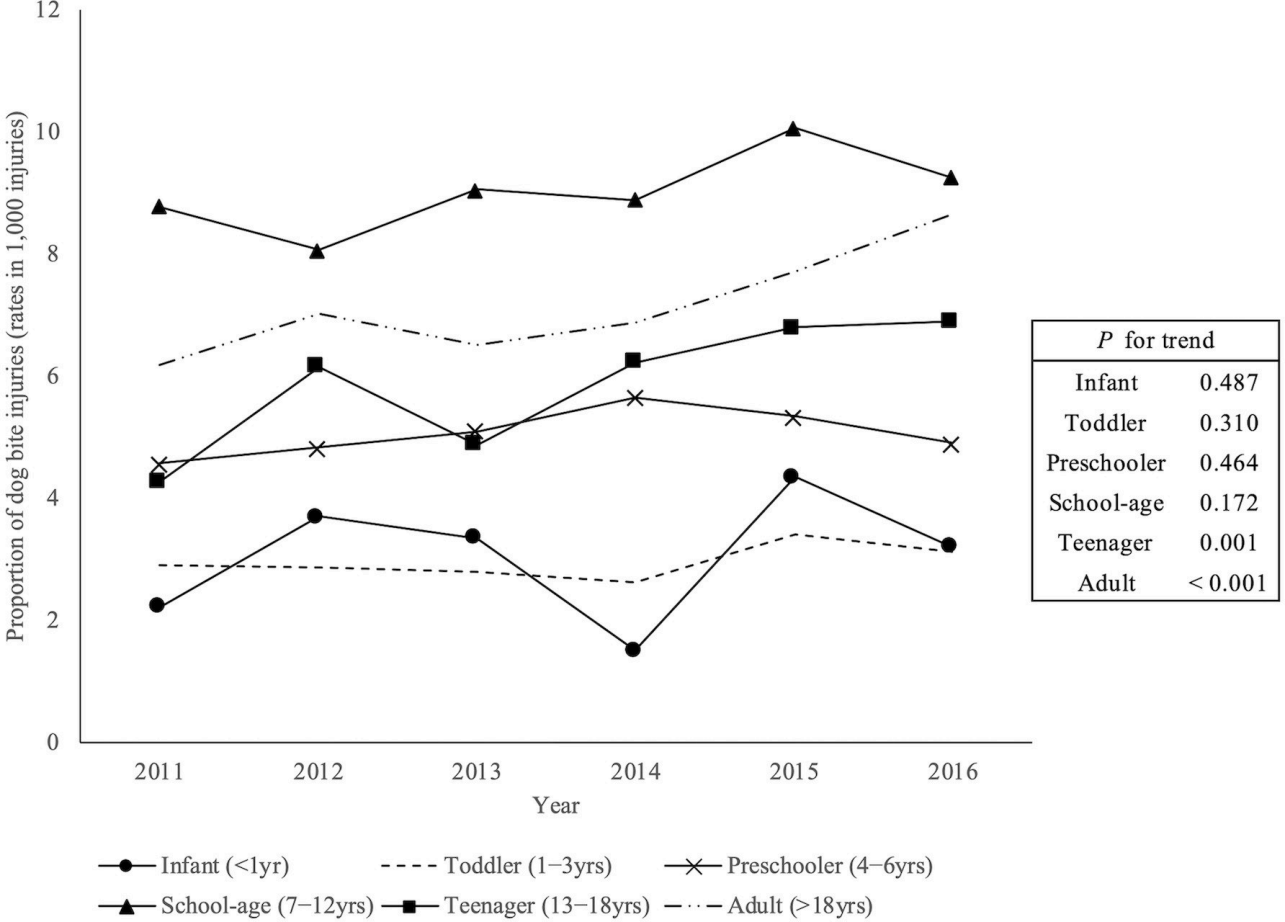
Age-specific trends of dog-bite injuries from 2011 to 2016. An increasing trend was observed for teenagers and adults, but there were no significant increasing trends in rates for any other age groups.

The annual rate of dog-bite injury per 1,000 injured patients increased throughout the study period (5.6 (2011), 6.3 (2012), 5.9 (2013), 6.2 (2014), 7.1 (2015), and 7.6 (2016)) (*P* for trend < 0.001). In the sex-specific analysis, both males and females showed increases in the rate of dog-bite injury from 2011 to 2016 (Fig 2). Fig 3 shows trends in the age-specific rates of dog-bite injuries between 2011 and 2016. An increase in rates was observed for teenagers and adults (*P* for trend = 0.001 and < 0.001, respectively), but there were no significant increasing trends in the rates for the other age groups. The highest prevalence was for school-aged children of 7−12 years, and the next highest prevalence was for adults.

Table 1 shows the basic characteristics of dog-bite injury by age group. The rates of dog-bite injuries per 1,000 injured patients according to age group were as follows: school-age children (9.0) > adult (7.2) > teenager (5.9) > preschooler (5.1) > infant (3.1) > toddler (3.0). The majority of the patients were female (54.6%), especially among adults (58.0%). In children aged 12 years or younger, however, males were predominant, except for infants. The most common anatomical site of bites was the upper extremity (33.3%), followed by the head and neck (21.9%), the lower extremity (15.7%), multiple sites (3.2%), and the torso (0.9%). In children, however, the head and neck were the most common anatomical sites of bites. The most common type of injury was open wound (79.2%), followed by superficial injury (17.4%), fracture (0.7%), and amputation (0.2%). Most of the fractures and amputations occurred in adults (64/72 (88.9%) and 14/16 (87.5%), respectively). The admission rate was 3.7%, and there were three fatal outcomes (one in the ED and two during the hospital admission). All patients who died were elderly individuals older than 70 years of age (93 years in the ED and 73 and 79 years during the admission). Among the hospitalized patients, the median hospital stay was eight days and was longer in adults than in children. A total of 224 (2.3%) patients underwent surgery, and the highest rates of surgery were observed in toddlers (2.8%).

**Table 1:** Characteristics of dog-bite injuries by age group.

Characteristics	Total		Age groups of years											
			Infant (<1)		Toddler (1−3)		Preschooler (4−6)		Schooler (7−12)		Teenager (13−18)		Adult (> 18)	
	N = 9,966		N = 86		N = 598		N = 528		N = 940		N = 573		N = 7,241	
Age, median (IQR)	32	(16–51)												
Gender (male), No. (%)	4,520	(45.4)	40	(46.5)	359	(60.0)	307	(58.1)	525	(55.9)	249	(43.5)	3,040	(42.0)
Bite site, No. (%)														
Head and Neck	2,184	(21.9)	37	(43.0)	230	(38.5)	154	(29.2)	236	(25.1)	202	(35.3)	1,325	(18.3)
Torso	85	(0.9)	0	(0.0)	4	(0.7)	9	(1.7)	30	(3.2)	4	(0.7)	38	(0.5)
Upper extremity	3,314	(33.3)	23	(26.7)	205	(34.3)	143	(27.1)	220	(23.4)	155	(27.1)	2,568	(35.5)
Lower extremity	1,563	(15.7)	8	(9.3)	47	(7.9)	120	(22.7)	182	(19.4)	46	(8.0)	1,160	(16.0)
Multiple^a^	319	(3.2)	1	(1.2)	18	(3.0)	17	(3.2)	47	(5.0)	16	(2.8)	220	(3.0)
Unknown	2,501	(25.1)	17	(19.8)	94	(15.7)	85	(16.1)	225	(23.9)	150	(26.2)	1930	(26.7)
Wound characteristics, No. (%)														
Superficial	1,736	(17.4)	26	(30.2)	119	(19.9)	131	(24.8)	214	(22.8)	111	(19.4)	1,135	(15.7)
Open	7,892	(79.2)	57	(66.3)	463	(77.4)	384	(72.7)	704	(74.9)	455	(79.4)	5,829	(80.5)
Fracture	72	(0.7)	0	(0.0)	0	(0.0)	1	(0.2)	6	(0.6)	1	(0.2)	64	(0.9)
Amputation	16	(0.2)	0	(0.0)	0	(0.0)	0	(0.0)	0	(0.0)	2	(0.4)	14	(0.2)
Others / Unknown	250	(2.5)	3	(3.5)	16	(2.7)	12	(2.3)	16	(1.7)	4	(0.7)	199	(2.8)
ED disposition, No. (%)														
Discharge	9,587	(96.2)	86	(100.0)	576	(96.3)	511	(96.8)	908	(96.6)	557	(97.2)	6949	(96.0)
Admission	370	(3.7)	0	(0.0)	22	(3.7)	17	(3.2)	31	(3.3)	16	(2.8)	284	(3.9)
Death	1	(0.0)	0	(0.0)	0	(0.0)	0	(0.0)	0	(0.0)	0	(0.0)	1	(0.0)
Unknown	8	(0.1)	0	(0.0)	0	(0.0)	0	(0.0)	1	(0.1)	0	(0.0)	7	(0.1)
Mortality^b^, No. (%)	3	(0.0)	0	(0.0)	0	(0.0)	0	(0.0)	0	(0.0)	0	(0.0)	3	(0.0)
Hospital stay (days), median (IQR)	8	(5–15)			6	(4–9)	7	(5–10)	7	(6–10)	6	(4–10)	9	(6–16)
Surgery, No. (%)	224	(2.3)	0	(0.0)	17	(2.8)	6	(1.1)	17	(1.8)	9	(1.6)	175	(2.4)
Place (home/others), No. (%)														
Home	7,206	(72.3)	85	(98.8)	482	(80.6)	329	(62.3)	641	(68.2)	455	(79.4)	5,214	(72.0)
Road	908	(9.1)	0	(0.0)	26	(4.4)	47	(8.9)	81	(8.6)	49	(8.6)	705	(9.7)
Commercial facilities	610	(6.1)	0	(0.0)	33	(5.5)	55	(10.4)	59	(6.3)	21	(3.7)	442	(6.1)
Countryside / Sea / River	372	(3.7)	0	(0.0)	19	(3.2)	29	(5.5)	42	(4.5)	13	(2.3)	269	(3.7)
Public cultural facilities	261	(2.6)	0	(0.0)	18	(3.0)	31	(5.9)	46	(4.9)	12	(2.1)	154	(2.1)
Others / Unknown	609	(6.1)	1	(1.2)	20	(3.3)	37	(7.0)	71	(7.6)	23	(4.0)	457	(6.3)
Place (indoor/outdoor), No. (%)														
Indoor	6,028	(60.5)	82	(95.4)	445	(74.4)	261	(49.4)	535	(56.9)	412	(71.9)	4,293	(59.3)
Outdoor	3,726	(37.4)	3	(3.5)	146	(24.4)	257	(48.7)	383	(40.7)	153	(26.7)	2,784	(38.5)
Unknown	212	(2.1)	1	(1.2)	7	(1.2)	10	(1.9)	22	(2.3)	8	(1.4)	164	(2.3)
Photoperiod, No. (%)														
Long day (April-September)	5,544	(55.6)	34	(39.5)	323	(54.0)	305	(57.8)	544	(57.9)	259	(45.2)	4,079	(56.3)
Short day (October-March)	4,422	(44.4)	52	(60.5)	275	(46.0)	223	(42.2)	396	(42.1)	314	(54.8)	3,162	(43.7)
Dog owners, No. (%)														
Household	3,144	(31.6)	48	(55.8)	253	(42.3)	112	(21.2)	244	(26.0)	212	(37.0)	2,275	(31.4)
Relatives	84	(0.8)	0	(0.0)	16	(2.7)	10	(1.9)	11	(1.2)	6	(1.1)	41	(0.6)
Neighbor / Friend	495	(5.0)	2	(2.3)	17	(2.8)	28	(5.3)	70	(7.5)	22	(3.8)	356	(4.9)
Stranger	851	(8.5)	1	(1.2)	34	(5.7)	63	(11.9)	102	(10.9)	31	(5.4)	620	(8.6)
Unknown	5,392	(54.1)	35	(40.7)	278	(46.5)	315	(59.7)	513	(54.6)	302	(52.7)	3,949	(54.5)

a Multiple wound was defined as a combination of one or more of the anatomic sites.

b Mortality was defined as death in the ED or during hospitalization.

**Fig 4:**
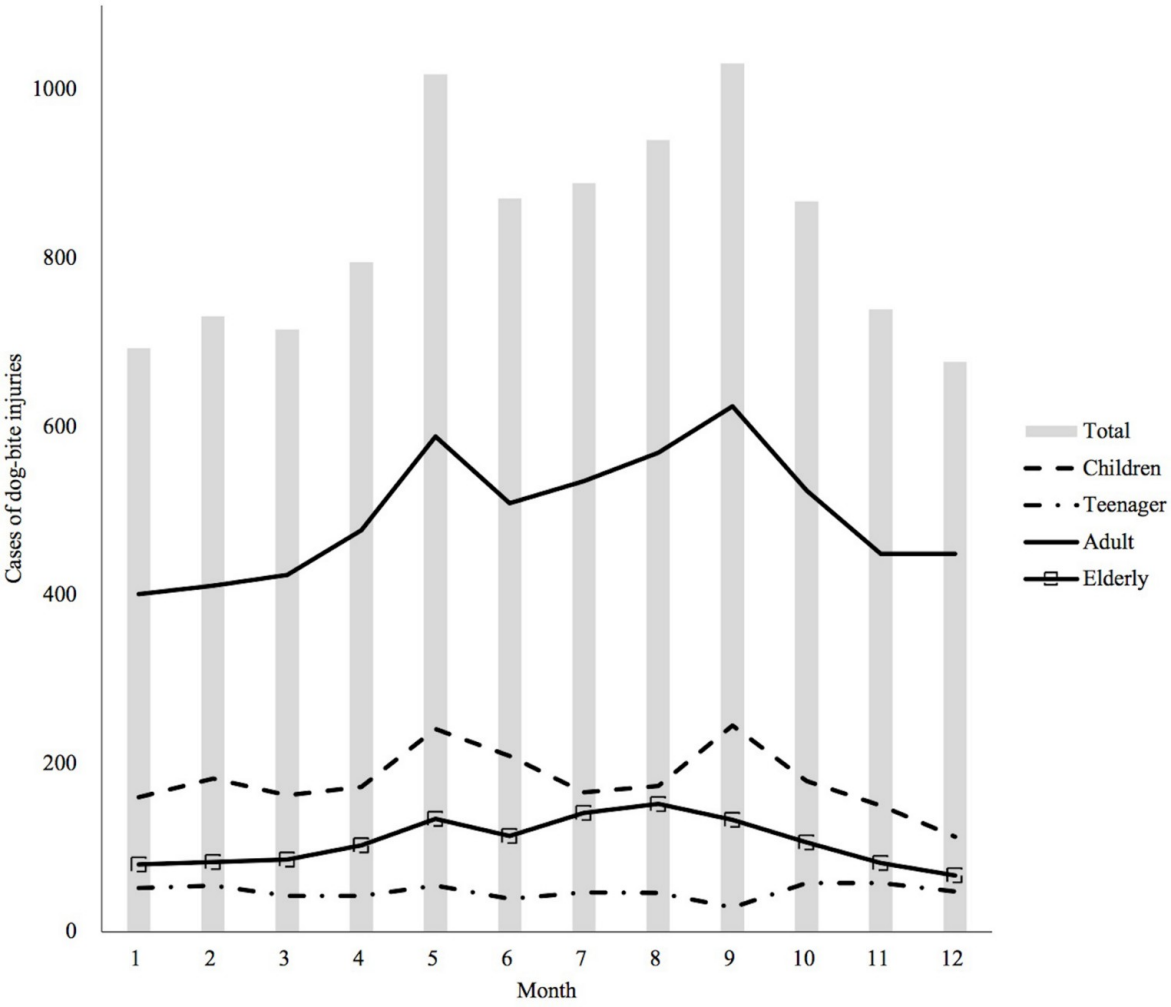
Monthly variation of dog-bite injuries.

The majority of dog-bite injuries occurred at home (72.3%) and indoors (60.5%). In most age groups, dog-bite injuries occurred indoors, but almost half of the preschoolers (48.7%) were injured outdoors. In the case of dog owners, family members were the most common (31.6%), and preschoolers (11.9%) and school-age children (10.9%) were more likely than the other age groups to be bitten by a stranger’s dog. Fig 4 shows the monthly variation in the dog-bite injury incidence. There were two peak incidences (May and September); particularly in the adults (19–59 years) and the children (0–12 years), these peaks were prominent. Dog-bite injuries occurred more during the long-day period (April-September) than during the short-day period (55.6% and 44.4%, respectively). However, infants and teenagers had more dog-bite injuries during the short-day period (60.5% and 54.8%, respectively) (Table 1).

### Risk factors associated with significant dog-bite injury

Table 2 shows the characteristics of significant dog-bite injury compared to non-significant injury. Among 9,966 patients, 489 (4.9%) had significant injuries. The results of multivariable logistic regression analysis are summarized in Table 3. Compared with adults (19–59 years), elderly people (≥ 60 years) were 2.7 times more likely to have significant injuries (95% CI: 2.15–3.39). Bite sites were also associated factors for significant injury (adjusted ORs [95% CI]: 2.63 [1.96–3.55] for head and neck, 1.73 [1.32–2.26] for the upper extremity, and 4.37 [2.96–6.45] for multiple sites). Outdoor injuries were 2.71 times more likely to be significant injuries than indoor injuries (95% CI: 2.20–3.34]). In the case of dog owners, relatives or neighbours/friends were riskier than owners in the household (adjusted ORs [95% CI]: 2.37 [1.09–5.17] for relatives and 1.68 [1.13–2.51] for neighbours/friends).

**Table 2:** Characteristics of dog-bite injury by severity.

Characteristics	Severity of dog bite-related injury				*P*-value
	Significant^a^ (n = 489)		Non-significant (n = 9477)		
Age, median (IQR)	46	(22–62)	32	(16–50)	**< 0.001**
Gender (male), No. (%)	207	(42.3)	4,313	(45.5)	0.169
Bite site, No. (%)					**< 0.001**
Head and Neck	123	(25.2)	2,061	(21.8)	
Torso	4	(0.8)	81	(0.9)	
Upper extremity	183	(37.4)	3,131	(33.0)	
Lower extremity	39	(8.0)	1,524	(16.1)	
Multiple	49	(10.0)	270	(2.9)	
Unknown	91	(18.6)	2,410	(25.4)	
Injury place, No. (%)					0.134
Home	344	(70.4)	662	(72.4)	
Road	46	(9.4)	862	(9.1)	
Commercial facilities	27	(5.5)	583	(6.2)	
Countryside / Sea / River	29	(5.9)	343	(3.6)	
Public cultural facilities	9	(1.8)	252	(2.7)	
Others / Unknown	34	(7.0)	575	(6.1)	
Injury place (indoor / outdoor), No. (%)					**< 0.001**
Indoor	188	(38.5)	5,840	(61.6)	
Outdoor	293	(59.9)	3,433	(36.2)	
Unknown	8	(1.6)	204	(2.2)	
Dog owners, No. (%)					**0.001**
Household	125	(25.6)	3,019	(31.9)	
Relatives	8	(1.6)	76	(0.8)	
Neighbor / Friend	38	(7.8)	457	(4.8)	
Stranger	39	(8.0)	812	(8.6)	
Unknown	279	(57.1)	5,113	(54.0)	

^a^ significant injury was defined as the patient’s death, hospitalization, surgery, or diagnosis of fracture or amputation.

The bold texts indicate P-value less than 0.05.

**Table 3:** Odds ratios (ORs) for the risk factors associated with significant dog bite-related injuries.

Characteristics	Total, No	Significant injury^a^		Unadjusted		Adjusted	
		No.	%	OR	95% CI	OR	95% CI
Age group (years)							
Children (0–12)	2,152	88	4.1	1.01	0.79–1.30	0.90	0.70–1.16
Teenager (13–18)	573	22	3.8	0.95	0.61–1.48	0.94	0.60–1.48
Adult (19–59)	5,960	241	4.0	1.00		1.00	
** Elderly (≥ 60)**	1,281	138	10.8	2.87	2.30–3.57	**2.70**	2.15–3.39
Bite site							
** Head and Neck**	2,184	123	5.6	1.58	1.20–2.09	**2.63**	1.96–3.55
Torso	85	4	4.7	1.31	0.47–3.65	1.41	0.50–4.00
** Upper extremity**	3,314	183	5.5	1.55	1.20–2.00	**1.73**	1.32–2.26
Lower extremity	1,563	39	2.5	0.68	0.46–0.99	0.58	0.39–0.86
** Multiple**	319	49	15.4	4.81	3.32–6.95	**4.37**	2.96–6.45
Unknown	2,501	91	3.6	1.00		1.00	
Injury place (indoor / outdoor)							
Indoor	6,028	188	3.1	1.00		1.00	
** Outdoor**	3,726	293	7.9	2.65	2.20–3.20	**2.71**	2.20–3.34
Unknown	212	8	3.8	1.22	0.59–2.51	1.29	0.62–2.67
Dog owners							
Household	3,144	125	4.0	1.00		1.00	
** Relative**	84	8	9.5	2.54	1.20–5.38	**2.37**	1.09–5.17
** Neighbor / Friend**	495	38	7.7	2.01	1.38–2.93	**1.68**	1.13–2.51
Stranger	851	39	4.6	1.16	0.80–1.68	0.93	0.63–1.37
Unknown	5,392	279	5.2	1.32	1.06–1.64	1.21	0.96–1.54

^a^significant injury was defined as the patient’s death, hospitalization, surgery, or diagnosis of fracture or amputation.

The bold texts are statistically significant findings.

## Discussion

As the proportion of households with dogs in Korea is increasing, it is important for healthcare providers and families to be aware of the frequency and severity of dog-bite injuries. This study evaluated the trends and characteristics of dog-bite injuries in Korea and analysed the risk factors associated with significant dog-bite injuries using a nationwide injury surveillance database. Dog-bite injuries were on the rise in Korea, which accounted for 0.6–0.8% of all injured patients. Of these patients, 3.7% needed hospitalization, and 2.3% underwent surgery. Among elderly individuals aged over 60 years, injury to multiple anatomic sites, being bitten outdoors, and being bitten by a relative’s dog were strongly associated with significant dog-bite injury.

From 2011 to 2016, ED visits for dog-bites were increasing and the proportion of dog-bite injuries per 1,000 injuries were also increasing. Although there were more participating hospitals since 2015, the number of dog-bite injuries per hospital was also increasing. A previous study in the United States reported that there was no significant difference in the incidence of dog-bite in the USA between 1994 and 2003 [10]. We are not able to know the annual incidence of dog-bites in Korea in this study. However, from the results of this study, we can presume that dog-bite injuries in Korea are growing as a public health problem.

Approximately 4.5 million people are bitten by dogs every year in the United States, and approximately 1 in 5 people bitten by a dog require some form of medical attention [10]. A previous report in the United States reported that the dog-bite injury rate was highest for children aged 5−9 years and decreased with increasing age [11]. In this study, the highest proportion of dog-bite injuries was reported in school-aged children of 7−12 years, which is similar to the findings in the US. However, the second highest rates of dog-bite injury occurred in adults over 18 years of age. In addition, dog-bite injuries in teenagers and adults but not children were on the rise. Although the reasons for this finding are not known, we can speculate that this difference may be caused by the reduced number of children playing outdoors in the modern era. Females were more likely to be bitten than males in this study, which is different from previous studies from the US and UK [11,12]. In the age-specific analysis, females were dominant among teenagers and adults, and males were dominant among children aged 12 years or younger, except for infants. A previous study in Italy reported that the distribution of bite incidences among males and females was significantly different in adults, with 66% being males and 34% being female [13]. As such, the age- and gender-dependent differences in the incidence of dog-bite injury may vary from country to country. This finding may be explained by the variety of cultures in terms of dog ownership and differences in dog breeds among countries.

Similar to other studies [11,12,14–16], the most common site bitten by dogs was the upper extremity, and head and neck injuries occurred more often in children than adults in our study. After ED treatment, 3.7% of patients needed hospitalization, and 2.3% of patients underwent surgery. Previous studies in the US reported that the hospitalization rate for dog-bite injuries was 4% [17] and 1.8% [11]. There were three fatalities during the study period. All patients were older than 70 years. Older age and complicated comorbid diseases may have contributed to these deaths. More than 70% of victims were bitten at home, but preschoolers were more likely to be bitten in other places, such as commercial facilities, than at home. In addition, preschoolers were more likely than the other age groups to be bitten outdoors and by a stranger’s dog. These results can be explained by the fact that young children, such as preschoolers, have exploratory behaviour and small bodies; therefore, they are especially vulnerable to dog bites outside.

Concordant with the findings of previous studies [17–19], a peak incidence of dog-bite injuries was reported during the warm season. The monthly distribution of dog-bite injury showed two peak incidences, May and September. In Korea, May and September have the best weather for going outside. Therefore, the peak incidence of dog-bite injury during the warm season may be explained by the fact that children and dogs tend to play outside during warm months, increasing the chances of encountering each other. In age-specific analysis, this finding was not observed in infants and teenagers. This difference could be because infants are usually in strollers when they go out, and there are few chances to encounter dogs. Korean teenagers are also similar because they are usually indoors (e.g., private educational institutes) for their study during warm months.

Previous reports in the US showed that the hospitalization rate after dog-bite injuries was highest for children younger than 5 years old and adults older than 65 years old [1]. In our study, significant dog-bite injury, including hospitalized cases, was associated with older age, especially in adults older than 60 years old. Complicating comorbid diseases, such as diabetes mellitus, could be more frequently observed at older ages, and such patients may have an increased risk of infection. A previous study reported that injury to the head, upper extremities or multiple anatomic sites is a risk factor for hospitalization after dog-bite injuries [20]. The results of our study were similar to those results. Compared to a single anatomical site, multiple anatomical sites had a stronger association with significant dog-bite injury. Multiple anatomical sites may mean that several dogs were involved or a wilder dog bit the patient, which implies that the patient is more likely to be more severely injured. Among single anatomical sites, the head, neck and upper extremity were associated with significant dog-bite injuries. Injuries involving the head and neck often require reconstructive procedures and can lead to disfiguring scars and long-term treatment. Several studies have shown an increased risk of infection in animal bites to the upper extremities compared to other anatomic sites [7,21,22]. As with previous studies [11,14,23], most victims in this study were bitten by a known dog. Brogan TV et al. reported that large dogs familiar to the child are usually involved in severe dog bites [24]. Our study showed that if the dog owner is a relative, neighbour or friend, the victims are more likely to have a significant injury than if the dog owner is stranger. Considering the time spent with dogs, people have more chance to bitten by known dogs than strangers’ dogs, because it is obvious that people usually encounter strangers' dogs for a moment while spending more time with known dogs. However, the bites by known dogs except own dogs were less frequent than the bites by strangers’ dogs. Moreover, injuries by known dogs were more serious than injuries by strangers’ dogs even though they are less frequent. This finding may be because the victim feels comfortable with the relative’s, neighbour’s or friend’s dog and is less vigilant, but the dog may not be familiar with the victim.

This study has several limitations. First, the characteristics of the dog-bite injuries were analysed based only on the pre-registered items in the EDIIS registry because this study is a retrospective cross-sectional study. Therefore, other potentially important data, such as dog size, dog breed, and rabies after dog bites, were not available, and these factors might affect the outcomes as potential confounders. Second, although EDIIS is a national injury database, not all EDs in Korea were included in the EDIIS. Based on 2014, the EDIIS had collected information from 20 EDs compared to the National Emergency Department Information System (NEDIS) which had collected information from 146 emergency medical centers, including all regional, specialized, and local emergency medical centers. This EDIIS data represents approximately 10% of injured patients data registered to the NEDIS (156,441 data in EDIIS and 1,522,348 data in NEDIS, respectively) [25]. Additionally, these EDs which participated in the EDIIS provide a relatively higher level of emergency medical services (level 1 or 2). Because of this selection bias, the results of this study may have a possibility of overestimation or underestimation. Third, we defined significant injury as fracture, amputation or death and the need for surgery or hospitalization in this study. Tendon or nerve injury and severe wound infection are also considered significant injuries. However, we could not extract these data from the database because this study is a retrospective study. Therefore, there is a possibility that some patients classified as having a non-significant injury according to the author’s definition actually have serious injuries from a clinical viewpoint.

## Conclusions

Our findings showed that ED visits for dog-bites in Korea are increasing, especially above youth age, and that injuries by a relative’s, neighbour’s or friend’s dog may be more dangerous than injuries by a stranger’s dog. The epidemiologic characteristics of dog-bite injury may vary according to cultural and socioeconomic differences. Therefore, each regional or national study is important for assessing country-specific differences in dog-bite injuries. Further studies should be performed to establish prevention strategies for reducing the incidence of significant dog-bite injuries in Korea. To decrease the incidence of dog-bite injury in Korea, preventive strategies that target not only children but also adults may be needed.
